# Some Practical Points in Regard to Methods in Construction of Crown and Bridge Work

**Published:** 1892-03

**Authors:** G. W. Melotte

**Affiliations:** Ithaca, New York


### Some Practical Points in Regard to Methods in Construction of Crown and Bridge Work.

BY G. W. MELOTTE, D.D.S., ITHACA, NEW YORK.

A talk given before the Ohio State Dental Society, Columbus, Dec., '91.

*Mr. President and Gentlemen of the Ohio Dental Society:*—Owing to the brief time allowed me, I shall only be able to touch upon a few points involved in the construction of crown and bridge work.

Gold, in holy writ, stands for truth, and for all the metals it must ever stand as the most useful in the construction of this new class, of gold restorations or bridge work, together with its companion, platinum.

It is curious to note the readiness with which pure gold and platinum can be welded by pressure.

Two pieces of pure gold and platinum having clean surfaces, perhaps one-half inch in width, and two in length, can be readily welded by grasping them in the pliers and bringing the pieces to a red heat over a Bunsen burner, then quickly passing them through the rolling mill, and the result will be the same perfect union of the gold and platinum that can be obtained in the manipulation of gold foil.

With this combination of metals the backing or lining of porcelain teeth can be accomplished with most perfect results. A tooth of light shade owing to its translucency, can be given a beautiful yellow tint by placing the gold side next to the tooth, or a gray tint will be produced by placing the platinum side in the same position; while the cutting edge or lower portion can be changed by using them in combination.

Pure gold, or any carat of gold ordinarily used in crown or bridge work, in making bands or collars, can be united or welded without the use of solder.

A creamy paste of borax and water is made by rubbing the borax in water on a slate or ground glass, and is applied to the surfaces of the metal; then with a soft flaring flame of the blow pipe the entire band is brought to a perfect red heat, when a concentration of the flame will produce a slight surface fusion or welding. It will be understood that in this welding the edges are lapped. The advantage of thus uniting metals without solder is, that the carat of gold is not lowered at the point of contact, rendering the entire band more malleable and therefore more readily adjusted to the natural crown, and less liable to melt in future heating. Should a stretching of the band be found necessary, it can be done on the horn of an anvil at the point where the lap is made.

I learned this method of welding from Dr. Bing, while visiting him.

We will now construct a bridge to supply the loss of the first and second bicuspid and molar, right side, lower jaw.

For the anterior anchorages or abutments we have a sound cuspid and the root of the lateral incisor; posteriorly, we have a sound molar. Many bridge workers would amputate the crown of the cuspid, making a Richmond crown; but objections are often raised by patients to such heroic practice; and to avoid cutting the tooth off, we will band it as is illustrated in "American System of Dentistry," Vol. 2, page 939; or see my article in "Cosmos" December, 1886, on "Crown and Bridge Work". Upon the lateral root may be placed a Richmond crown; these when united will form a perfect anterior anchorage. The molar which is to form the posterior anchorage, having no occlusion with an upper tooth, may be banded and capped. See as above.

The anchorages or abutments having been constructed and placed in their positions, the next very important consideration is taking the bite for the occlusion of the teeth to be supplied. Modelling or impression compound, made plastic, is placed along the ridge between the abutments, and the patient is then directed to close the mouth, using care as to correctness of the closure; cold water may then be applied to the compound with a sponge or napkin, for the purpose of cooling or hardening the material. Remove the compound, and with a knife cut away from the buccal and lingual sides, leaving just enough to give the impression of the upper teeth, and leaving the gold anchorages exposed as much as possible, consistent with the retention of the compound impression, when replaced, avoiding the lapping of the compound on to the grinding surface of the gold crown anchorage. An impression of the whole is now taken in plaster, which includes all the anterior and posterior teeth. When the plaster is set quite hard, remove the impression, and if the segment of modelling compound should separate in the removal it can be readily replaced ; and also it is important to see that the anchorages are in their proper positions.

We will next place two dowels made of ordinary pins in the impression of the central and cuspid, press the points into the plaster sufficiently to hold them in an upright position, then with cutting pliers cut the pins, leaving them about one-half inch in length. The impressions of the crowns of the teeth are then filled with fusible metal, melting at about 150 degrees. The balance of the impression is then varnished, the anchorages filled with investment material, Plaster of Paris and Marble Dust. When hard, the impression cup is first removed, and the plaster is cut away from the metal teeth, care being taken not to mar the segment of modelling compound.

The advantage of the fusible metal is that it obviates the danger of defacing the teeth not used as anchorages, but necessary in the occlusion. The modelling compound will be seen on the model in the same relation that it was before taking the impression in the mouth. An impression of the antagonizing teeth in the upper jaw can now be taken in plaster, and the fusible metal poured into the impression at once, and as soon as cool it can be removed, thus giving a perfect representation of the upper teeth, which are to be placed into the imprint of the modelling compound, and then adjusted in an articulator. By this method a very perfect articulation of the teeth can be obtained, which is of great importance to bridge work.

Time will not admit of my dissecting the methods involved in working dummies, which are to form the bridge.

A member. Do you band before you put the fusible metal in?

*Dr. Melotte* :—I do not. It would be well to do it, but I have not got time, as a rule, to do that. Perhaps it would be well for a man to do it when he first commences to do this kind of work.

A member. Will you please tell us how you weld gold?

*Dr. Melotte:*—"The Lord breathed into man the breath of life." I use some of it in connection with the blow-pipe, and by coating the surface with borax you are enabled to bring it up to the heat of fusion. The molecules of the upper layer to the lower layer will come in contact with heat and readily unite. If you are not careful you will spoil your band, so it is well enough to take a good many pieces of gold and lap them. In doing this work it is well to have a blacksmith's heat, which is a general heat, heating your band up thoroughly before you concentrate the heat, otherwise it is better perhaps to point the flame a bit—heating it up until the material is ready to melt, then you get your welding. You may spoil a number of bands of gold before you are able to succeed, but you will be surprised how well you can do it if you learn to emit a nice flame with the blow-pipe.

A member. Why do you use shellac on porcelain?

*Dr. Melotte:*—Because in burning, it carbonates and gives you a layer of charcoal next to the porcelain. It protects the porcelain more perfectly than it is protected with the ordinary investment of plaster of Paris.

*Dr. Harroun:*—I am in the habit of coating the surface of a tooth to prevent the plaster of Paris from melting. It forms a curtain there, so to speak, prevents the plaster from shrinking down, and forms a roughness on the surface.

*Dr. Melotte:*—That point was suggested to me by a dentist of Utica in combining the porcelain and gold. He told me that was the course he pursued. I find it is a nice thing to do. I am conscious of the fact that with porcelains you can not be too careful to keep them from contact. Considerable expansion and contraction take place when the molecules are under the excitement of returning to their primitive condition. Of course there is great danger of breaking. Dr. Starr suggested that pieces of mica be put between the porcelains we use. In olden times it was suggested that a piece of tissue paper or writing paper put in between would prevent breaking. I think mica is a very good thing indeed for this purpose. We should see that the porcelains are not in contact when they are invested.

I have reached a point now where I have constructed my abutments. I call your attention to one of the most important points connected with the work, and yet one of the simplest—taking the impression. If this tooth (illustrating) leans toward the anterior teeth, or if there is a wedge-shape or straight space, you may have trouble from breaking of the plaster, making your impression imperfect. To obviate that I take a piece of modelling compound and press it with my fingers between the teeth. I remove it, put it into cold water, then with a knife I trim on the buccal and lingual surfaces, leaving them a little narrower on the top. Before placing it back I warm the surface and direct the patient to bite down on to it, leaving an impression of the occluding teeth. Be careful to see that the patient bites correctly. Now, remove the piece again and trim so that the modelling compound will come in contact with the anterior and posterior portions of the band, but not to cover any more than you can help. You trim it and replaced it. It is in position. The patient has bitten down upon it. You may let them try it again if you are certain they will bite in the same place. The imprint of the cusps are there. Now you take the impression in plaster, the modelling compound being in position. You take your plaster and if the compound remains in the mouth and does not give way with the plaster, remove it and put it in its place. Now, in taking an impression I find that the imprint of this tooth is in the impression. There are few men who prac tice making impressions with bands on, preparatory to making a restoration or bridge; they take impressions in modelling compound. They may be experts in that particular line, and they may succeed in a small piece of bridge work, and may with their method save time, but I would not attempt to do it. A gentleman said to me a short time ago, "It takes so long to remove a plaster impression from the model," and that is one of the reasons why he does not use it. Then again, sometimes patients object to certain things you want to do. My patients do not object because I do just as I have a mind to. As a rule I do, or else I do not do the work.

I have taken the plaster impression and am about to pour into it plaster of Paris to make my model upon which my bridges are constructed. I take two pins and place them into the impression of two of these teeth a little ways apart. The heads are standing up, and the impression is before you, and you are looking down into the imprints of the teeth. I take the pins, place them in the cutting edge of the teeth in this case, then I take some little bits of fusible metal and cut it off with excising forceps and place it into the imprint of the teeth, and with a puff of the blow-pipe diffuse the metal and jar it down, and then you have got the tips of the teeth like that which you see represented on the board. Dr. Templeton has three or four combined tips that I removed from a case while he was with me. He will show these tips to you at my clinic this afternoon. I will take an impression of some one's mouth and show you the method of making these tips. You cut off the ends of the pins and leave the dowels around them, leaving the pins one-half or one-quarter of an inch in length, then fill up the model with investment material, plaster of Paris and marble dust. One-third marble dust and two-thirds plaster of Paris is about the formula. You have finished the model previous to pouring fusible metal in there. Your plaster is hard, you cut away and down on to these teeth, cutting away the plaster impression you cut the tips. You are not endangering or marring them. I have got to a place now where I find my modelling compound is in the same position with the cusps upward (this being a lower case) that it was when the patient left the imprint. You readily see that. When I have taken an impression of the occluding teeth (and I take with plaster) I pour in fusible metal quickly. If I do not get a perfect occlusion I can pour it several times ; I get it quicker than it takes the plaster to set.

A member. Will you tell us how you make a dummy?

*Dr. Melotte:*—I will state first what happened in my office. My assistant was working for an elderly gentleman and he got to the point of adding the dummy, and he turned to me and said,

"What shall I do with the dummy?" The man looked up at me with daggers in his eyes but the young man gave him to understand that he did not mean him.

I do not know how they came to be called dummies unless they were considered dummies who worked at them first.

I will make the first dummy and shall prefer to take an ordinary cuspid tooth, and in grinding it I may find it is too long from the pins up to the cutting edge. Dr. Richmond showed me this method of grinding a cuspid tooth to keep the proportions perfect, that is, to grind from the lingual toward the labial surface, as perhaps you are able to see this tooth (illustrating). You take a tooth that is blunt on the end, the advantage is that you make it upon the porcelain, so that when you add gold you have got a better looking tooth than you would otherwise have; then, besides, you first put a piece of backing on, and this backing and the tooth are ground on the surface, the backing being beveled with the porcelain. You then take a piece of thick gold and where you want great strength on the point and desire to prevent the teeth from breaking, I should use gold that has about 5 per cent. of platinum, using No. 23 or 24 and let it extend about a line above the cutting edge, wax it, and it would be ready for investment. You take a die and swage out a bicuspid, take one of the bicuspid dies, swage out a piece of gold, place it on, bevel toward the outer cusp, so that it will fit on to the tooth in the manner in which I hold this, wax it in place and invest it, varnishing it at first. Invest in plaster of Paris, marble dust or sand, then after the investment has set, dry it out, melt out your wax, add your gold carefully; you may put in tips of gold coin or fillings, fill it up with fillings and solder, and in that way you have got your dummy made for your first bicuspid. Proceed with the next and with the molar in a similar manner, and you have your dummies, which, of course, you have tried on and arranged with a view to the occluding teeth. Of course, this work needs care and time, and I can not find language to describe to you definitely just how to proceed. There is an important point I want to make, and that is that each one of the dummies should be ground, the porcelain and the gold, so that they will touch the ridge, and especially in those cases where there are any bridges which failed to have touched, because the tissues will kindly form around the points of contact and you need not be much afraid of inflammation, and if you get a little hypertrophy you can allay it as well with a saturated solution of salicylic acid as any thing, and after a little the gum will kindly form and it comes up around rather than shrinks away from the dummy.

During the Doctor's remarks he made use of illustrations, the absence of which detracts materially from the clearness of the Doctor's verbal description, but to make up for it, reference has been made above to his articles on the subject, illustrated in the "American System of Dentistry" and "Cosmos".

In the afternoon the Doctor gave a clinic, demonstrating the methods referred to in his address, but laboring under the usual disadvantages incident to the want of the many conveniences of his own office.

